# A qualitative study of patient’s experiences of receiving alcohol interventions according to the 15-method in psychiatric care

**DOI:** 10.1186/s12888-025-07553-1

**Published:** 2025-10-28

**Authors:** Jennie Sundbye, Anders Hammarberg, Joar Guterstam, Sigrid Salomonsson, Sara Wallhed Finn

**Affiliations:** 1 Centre for Psychiatry Research, Department of Clinical Neuroscience, Karolinska Institute & Stockholm Health Care Services, Tomtebodavägen 18A, Region Stockholm, Solna, 17176 Sweden; 2 Department of Global Public Health, Karolinska Institute, Solnavägen 1E, Stockholm, 11364 Sweden

**Keywords:** Alcohol, Alcohol use disorder, Psychiatric disorders, Psychiatric outpatient clinics, Comorbidity, Screening, Integrated treatment, Qualitative, Implementation, Normalization process theory

## Abstract

**Background and aim:**

Comorbidity between psychiatric disorders and alcohol use disorders (AUD) is common. Swedish national guidelines recommend integrated treatment for both disorders, however, compliance to the guidelines is low. To facilitate uptake, health care staff at psychiatric units in Region Stockholm, Sweden, have participated in training and implementation of the 15-method for screening and treatment of AUD. Few studies focus on the patient perspective of interventions for AUD in psychiatric care. The aim of this study was to investigate how patients in psychiatric outpatient care perceived receiving interventions according to the 15-method, within the context of a large-scale implementation project.

**Methods:**

Qualitative study with data collected through individual interviews involving 15 adults who had Alcohol Use Disorder Identification Test (AUDIT) score >6 for women/ >8 for men and contact with a psychiatric unit whose staff had taken part in the implementation project of the 15-method. The interviews were analyzed with reflexive thematic analysis using the four constructs of the Normalization Process Theory; coherence, cognitive participation, collective action and reflexive monitoring, as a framework.

**Results:**

All participants had received alcohol screening, four had received the assessment in the second step in the 15-method, three received pharmacotherapy, however, none reported receiving the psychological treatment. A majority found it natural to discuss alcohol within the psychiatric care setting and described that these conversations led to increased awareness about their hazardous alcohol consumption (*coherence*). Alcohol use was perceived as a sensitive topic and staff attitude was important to create a working alliance (*cognitive participation*). Conversation about alcohol could evoke behavior change but few participants had been offered alcohol interventions beyond screening (*collective action*) and expressed a wish to receive more support (*reflexive monitoring*). Treatment for psychiatric symptoms was described as conditioned to non-hazardous level of alcohol consumption (*coherence*), leading to active actions to become eligible for treatment (*cognitive participation*). Moreover, conditioning of care mainly led to short-term behavior changes (*reflexive monitoring*).

**Conclusions:**

Patients in psychiatric care are positive to receiving alcohol-interventions within this context, indicating that psychiatric care offers a window of opportunity to address comorbidity. However, future staff training and implementation initiatives should be tailored to the specific challenges in psychiatric care, such as conditioning of care.

**Clinical trial number:**

Not applicable.

**Supplementary Information:**

The online version contains supplementary material available at 10.1186/s12888-025-07553-1.

## Background

Alcohol use is highly prevalent worldwide, contributing to substantial health loss, disability and premature mortality [1, 2]. In Sweden, 16% in the general population self-report hazardous alcohol use [3] and 11,2% are estimated to fulfill the DSM-5 criteria for Alcohol Use Disorder (AUD) [4]. It is common that AUD and other psychiatric disorders co-occur [5] and the prevalence of hazardous alcohol use is markedly higher in adult psychiatric populations compared to the general population [6–9]. Hazardous alcohol use and AUD are associated with higher levels of psychiatric symptoms and higher incidence of suicide compared to psychiatric patients without AUD [10–12]. It is therefore important to offer patients with comorbidity interventions to reduce harm. International guidelines recommend that patients with co-morbidity should be offered simultaneous interventions [13] and similarly, the Swedish national guidelines, recommend integrated treatment for patients with co-morbidity [14]. However, compliance to the Swedish guidelines is low [15]. Studies on implementation of AUD interventions in psychiatric care suggest that several barriers exist, for example lack of knowledge and routines, time constraints, reluctance among staff to address alcohol out of fear of offending the patients, and stigmatizing attitudes [16, 17]. Few studies have focused on how patients perceive addressing alcohol use in psychiatric care. Data from Poland and the United Kingdom emphasize the importance of health care staff raising the topic of alcohol, as patients expressed a reluctance to do so due to stigma and the risk of losing access to treatment for psychiatric disorders [18, 19]. Moreover, the patients expressed a wish to discuss the association between their psychiatric diagnoses and AUD without the risk of negative consequences, like withdrawal of psychiatric medication [19].

To facilitate the implementation of local guidelines for the identification, assessment and treatment of AUD in adult outpatient voluntary psychiatric care, the management team for psychiatric care in Region Stockholm, Sweden, decided in 2020 to offer all staff training in a manual to treat AUD - “the 15-method”. The 15-method is a stepped care model for screening, assessment and treatment of AUD in non-specialized addiction settings, targeting patients who have hazardous alcohol use or mild to moderate AUD [20]. The 15-method was originally developed and evaluated in a randomized controlled trial in primary care. The trial showed that treatment in specialized addiction care was not superior to treatment with the 15-method in primary care, indicating that the 15-method is a promising approach outside of specialized addiction care [20–22]. According to regional guidelines, patients with Alcohol Use Disorder Identification Test (AUDIT) scores ≥ 18 for women/ ≥20 for men, indicating a more severe alcohol dependency, should be referred to specialized addiction care [23].

The 15-method consists of three steps, each evidence-based; 1. Screening with AUDIT and brief interventions [24] 2; assessment with a 30-minute feedback session [25] 3; four sessions based on cognitive behavioral therapy (CBT) and motivational interviewing, ‘Guided Self-Change’ [26, 27] and/or pharmacological treatment with disulfiram, acamprosate, naltrexone or nalmefene. Guided Self-Change consists of themes to facilitate behavior change such as goal setting, self-monitoring of alcohol consumption, identifying risk situations and problem solving [27]. For the present project the manual was adapted to psychiatric care: recommendations for AUD pharmacotherapy in conjunction with treatment for other psychiatric conditions; information on how alcohol can affect psychological health and recommendations to involve different professions in the delivery of the interventions. The manual recommends that the interventions for AUD and the psychiatric disorder are offered within the same treatment team but gives no specific guidance on how the work should be organized. The psychiatric care units offer both pharmacological and psychological treatments for psychiatric diagnoses, which should follow national guidelines according to each diagnoses [28].

For new guidelines to become embedded in routine care, effective implementation is vital. To understand the implementation process of the 15-method, it is important to study the patients’ perspectives of receiving these interventions. The Normalization Process Theory (NPT) is a well-established and well-used theoretical framework developed to understand the process of implementation, both from a health care provider perspective and end-users, such as patients [29–31]. In NPT the users´ actions and sense-making of the implementation is in focus, through four constructs: coherence, cognitive participation, collective action and reflexive monitoring. Using NPT as a framework, can provide concrete recommendation on how implementation can be improved.

The aim of this qualitative study was to investigate how patients in psychiatric outpatient care perceived receiving interventions according to the 15-method through the lens of NPT, within the context of a large-scale implementation project.

## Method

### Study design

Qualitative study with data collected via individual interviews. The study conformed to the Standards for Reporting Qualitative Research [32] and was approved by the Swedish Ethical Review Board (Dnr 2022-03977-01).

### Training in the 15-method and the BIC-intervention

From spring 2021 to autumn 2024, training in the 15-method was offered to all clinical staff at psychiatric outpatient units in Region Stockholm. Unit managers signed up their clinical staff for the training and approximately 360 clinical staff from 35 units participated. The training was completed via two four-hour long live video lectures which included role plays and group discussions. The training was delivered by authors AH and SWF and a clinical psychologist from specialist addiction care. To further support the implementation process, three to six clinical staff and one manager from each unit received training in the Building Implementation Capacity (BIC) intervention- a systematic implementation model focusing on identifying target behaviors, analyze barriers and facilitators for successful implementation, choose implementation strategies and try them out [33, 34]. The training was completed in four three-hour long workshops with lectures interspersed with time for the participants to plan their unit´s implementation of the 15-method.

### Participants and setting

We used a purposive sampling strategy and aimed for variation in age, gender, AUDIT scores, psychiatric diagnoses, psychiatric units and received steps in the 15-method.

Inclusion criteria were: ≥18 years of age; ongoing contact with a psychiatric outpatient unit whose staff had participated in the described training project, recall of at least one conversation about alcohol at the psychiatric unit after the unit took part in the training; hazardous alcohol use defined as AUDIT scores >6 for women/ >8 for men [23]. Exclusion criteria were ongoing psychotic episode; severe cognitive impairment; and non-fluent in Swedish. The exclusion criteria were assessed during the screening interview by the screeners who were registered nurses, assessing whether the participant had difficulties answering any of the screening questions or in other ways showed behaviours which could indicate psychotic symptoms or severe cognitive impairments. All participants gave informed consent to participate.

### Procedure

Posters and flyers with information about the study and contact details to study-coordinators were placed at the 21 psychiatric units which at the time had finished training in the 15-method and BIC-intervention, and clinical staff were asked to hand out flyers to patients who had received treatment corresponding to step three. Those who showed interest were telephone-screened for eligibility and thereafter scheduled for an interview.

Data was collected between January 2023 and April 2024 through individual interviews via telephone or in person, at the participant’s preference. With few recruited participants who had received step two and three in the 15-method, the screening for eligibility was extended, however, no further participants were included and the recruitment closed in August 2024. A semi-structured interview guide (Additional file 1) was developed by JS and SWF, focusing on the research aim and the four NPT constructs (Table 1).

**Table 1:** Core construct of the normalization process theory, applied on the 15-method

NPT Construct	Description	Application to the 15-method
*Coherence* - The meaningful qualities of a practice	How is a practice conceptualized by participants? How does it hold together in action?	How do the patients understand the steps in the 15-method? Do they share a common view of the purpose of the different steps? Do they understand how the intervention affects them personally? Do the participants grasp a potential value and benefit of the 15-method?
*Cognitive Participation* - Enrolment and engagement of individuals and groups	How do participants come to engage with a practice? How do they decide on engagement and the purposes that it serves?	How do the patients participate in the 15-method? What builds engagement and keeps the patients motivated to continue to participate?
*Collective Action* - Interaction with already existing practices	How do participants enact a practice? How are their activities structured and constrained?	How do the patients make the changes triggered by the different steps in the 15-method? What strategies to they apply to accommodate the new practice, i.e. reduced alcohol use?
*Reflexive Monitoring* - How practice is understood and assessed by actors implicated in it	How do participants appraise a practice? What are its effects of appraisal? How are they mediated?	How do the patients experience that their participation in the 15-method affects them and other people? Do the patients value participating in the 15-method as worthwhile over a longer period of time?

JS conducted all interviews which lasted between 31 and 68 min (mean 49 min). The participants were reimbursed with two cinema tickets. Interviews were audio-recorded and transcribed verbatim.

### Researcher characteristics

The research team included: a PhD student who is a registered nurse and M.Sc. in Public Health (JS); a clinical psychologist with a background in clinical addiction treatment and PhD in alcohol research (SWF); a licensed psychotherapist with a background in clinical addiction treatment and PhD in alcohol research (AH); a psychiatrist and addiction medicine specialist with a PhD in the addiction field (JG); and a clinical psychologist with a PhD in clinical psychology, experienced in leading implementation in health care (SS).

### Analyses

Data was analyzed with reflexive thematic analyses as described by Braun & Clark (2006). To familiarize with the material, transcripts were read circa 3–4 times each by both JS and SWF. Information power was decided to be sufficient according to established criteria [35]. Coding was deductive and mapped to the four constructs of NPT (Table 1). Initially, JS coded the data and thereafter discussed and refined the codes with SWF. In the next step, JS and SWF jointly discussed, developed and reviewed the sub-themes under each construct. Code labels and sub-themes were discussed and agreed upon in the wider research team. The transcripts and the analysis were completed in Swedish and thereafter the citations were jointly translated into English by JS and SWF. Nvivo (1.7.1) was used to assist data analysis.

## Result

### Participant characteristics

In total, 83 people were screened for eligibility, of whom 15 participants from nine different psychiatric units were eligible and included in the study (Table 2). Of those not included in the study; *n* = 37 reported conversations about alcohol at a psychiatric unit not part in the implementation project or before the staff training was completed; *n* = 25 reported AUDIT scores ≤ 6/8, and *n* = 6 due to other reasons, such as expressing interest in participating but could not be reached.

**Table 2:** Characteristics of participants

Name (fictive)	Age	Gender	AUDIT score	Highest completed education	Occupation
Max	18–29	None binary	13	High School	Unemployed
Andrea	18–29	Female	20	Elementary school	Student
William	18–29	Male	23	Elementary school	Disability compensation
Matilda	18–29	Female	28	High School	Sick leave
Hans	30–39	Male	14	High School	Sick leave
Susan	30–39	Female	18	University	Working
Johan	30–39	Male	19	University	Working
Wilma	30–39	Female	21	Vocational university	Sick leave
Fredrik	30–39	Male	22	University	Working
Karin	40–49	Female	12	Elementary school	Sick leave
Lydia	40–49	Female	15	University	Working
Marit	40–49	Female	21	University	Working
Lisa	40–49	Female	22	University	Working
Krister	40–49	Male	23	University	Working
Kristin	50–59	Female	16	High school	Sick leave

A majority were women, mean age 37 years and all but one were born in Sweden. Four participants reported AUDIT scores indicating hazardous alcohol use while eleven participants had AUDIT scores > 15, indicating probable dependence. The participants’ psychiatric diagnoses varied; affective disorders, anxiety disorders, neuropsychiatric syndromes, personality disorders and psychotic disorders. Most had more than one psychiatric diagnosis. Length of contact with the present psychiatric care unit varied from 1,5 months to 10 years, with a median of 2,5 years. The contact status varied from planned treatment, ongoing and completed, as did type of treatment – psychological, pharmacological and/or neuropsychiatric assessment or other psychiatric diagnostic assessments. Two participants had a parallel contact with specialized addiction treatment services.

### Type of alcohol interventions received

All included participants described that they had been screened for alcohol use during their contact with the psychiatric unit, corresponding to step one in the 15-method. The screening was completed by filling out the AUDIT questionnaire and/or giving samples for biomarkers for alcohol consumption, such as phosphatidylethanol (PEth) and/or carbohydrate-deficient transferrin (CDT). The screenings were followed by different types of conversations about alcohol. Participants generally gave vague descriptions of the alcohol interventions they had received, making it difficult to determine whether these matched the steps of the 15-method. Four participants recalled receiving the assessment procedure in step two and perceived it as feasible without elaborating further. Three participants described that they had received pharmacological treatment for AUD, corresponding to step three, while none reported receiving the psychological treatment ‘Guided Self-Change’.

The results are categorized in sub-themes around the core constructs of NPT (Table 3) and presented for each construct.

**Table 3:** Sub-themes by NPT constructs

NPT construct	Sub-theme
Coherence	• Alcohol assessment - a natural part of the psychiatric care process • Increasing awareness • Conditioned psychiatric treatment
Cognitive Participation	• Alcohol use – a sensitive topic • Active actions to receive psychiatric treatment
Collective action	• Left alone with demands to reduce alcohol use • Conversations have the potential to evoke change
Reflexive Monitoring	• Responses to conditioned psychiatric care • A wish for more integrated treatment • Relevance for the future

### Coherence

#### Alcohol assessment - a natural part of the psychiatric care process

Alcohol screening was described as an incorporated part of the regular psychiatric visits, mainly perceived as positive and that screening made sense, as alcohol use and psychiatric disorders were often perceived as associated. One participant discussed that it would probably be more difficult to have separate conversations about alcohol:

*…if I were to have my treatment with my therapist and then I would have separate conversations specifically about alcohol with someone else*,* then I don’t think… I wouldn’t have found it as easy to talk about it.* (Hans, 30–39 years)

In general, the participants perceived that the purpose of step one was to map in which situations they used alcohol, what triggered their alcohol use and consequences of it. A further purpose was to understand reasons behind high consumption, how it was associated to the psychiatric condition as well as assessing the level of their alcohol use as it could affect what treatment they would be offered.

*It has been about getting to the bottom of what the alcohol consumption and especially the excessive alcohol consumption comes from. Which factors that have led to it and simply how one can handle it*. (Johan, 30–39 years)

The AUDIT questionnaire was perceived as both easy and feasible, and repetitive and meaningless- conveyed as a statistical and non-personalized assessment procedure. Most of the participants had also been screened with biomarkers however, the views of biomarkers as a screening method were mixed and to some participants the purpose was unclear. Others discussed a lack of feedback on the results. The perceived purpose of the alcohol screening affected how the participants responded to the screening, elaborated in the sub-theme ‘Active actions to receive psychiatric treatment’.

#### Increasing awareness

The participants described that a consequence of the screening was an increased awareness that their alcohol consumption was high or hazardous.

*These conversations have made me… well sort of open my eyes a bit and realize how serious this is.* (Andrea, 18–29 years)

Conversations about why and when they used alcohol, instead of solely focusing on the level of consumption, was appreciated and led to further reflections around their alcohol use and increased awareness of the association between their psychiatric disorders and alcohol consumption.

#### Conditioned psychiatric treatment

Participants recollected receiving information on how alcohol use can affect the psychiatric treatment offered, but also information that hazardous alcohol use was not allowed during specific psychiatric treatments such as Cognitive Behavioral Therapy (CBT) for post-traumatic stress disorder (ptsd) or pharmacological treatment for attention-deficit / hyperactivity disorder (adhd) with stimulants. The participants conveyed a perception that psychiatric treatment was conditioned in relation to their level of alcohol use, where a hazardous consumption could lead to treatment being paused or not initiated.

*It has mostly been discussions about*,* what is a reasonable amount and how these PEth-tests work and it has been very much around that I could not receive other help with medication or anything else until I reduced my alcohol consumption*,* so it was a bit of a barrier to initiate my treatment… and that was something that I felt was quite a large source of irritation.* (Fredrik, 30–39 years)

### Cognitive participation

#### Alcohol use - a sensitive topic

Talking about one´s alcohol consumption was described as shameful and trying but also positive. Alcohol consumption was perceived to be a sensitive topic triggering feelings of embarrassment or shame, and in addition, some discussed that the clinical staff seemed uncomfortable when raising questions about alcohol. Talking about how much the participants drank or things that had happened under the influence of alcohol, could evoke feelings of guilt. A non-judgmental attitude from the clinical staff was associated with feeling safe to talk openly and acknowledging a problematic consumption.

*The risk is that one feels… guilt*,* that it imposes guilt upon you. I don’t think it has been like that. On the contrary*,* that it is acknowledged that it is harmful*,* that it is taken seriously. […] I think it took a little while for me to get there*,* to feel that it was ok to have this diagnosis* (note: alcohol-related diagnosis) *and to see it as the risk that it is.* (Lisa, 40–49 years)

However, some participants perceived that the clinical staff had a judgmental attitude, which made the participant feel uneasy and withdrew from further discussions about alcohol.

#### Active actions to receive psychiatric treatment

Due to the conditioning, participants took deliberate actions to ensure they were eligible for treatment, such as reducing alcohol use before giving blood samples or underreport alcohol use.

*I believe that is also why I actively avoid bringing it up*,* partly because it can affect my treatment options here*,* but also that it has become clear for me in those conversations that*,* this specific resource is not available here. Instead*,* I need to seek it out in other places and if I express a need for help with alcohol problems then that becomes a warning flag in the therapy I attend. (Matilda*,* 20–29 years)*

Three of the 15 participants had received pharmacological treatment for AUD, corresponding to step three. One had requested the treatment himself while one had started the pharmacological treatment at a specialized addiction unit. This patient conveyed she had to argue with the physician at the psychiatric unit to change type of AUD pharmacotherapy when she perceived that the effect of the first one decreased.

None of the participants recalled any discussions around different AUD pharmacotherapies and one participant described not having any follow-up visits for the AUD medication, instead he himself had to find out the most effective way to use the medication.

### Collective action

#### Left alone with demands to reduce alcohol use

Few of the participants recalled being offered interventions to support them in reducing their alcohol use. One participant described receiving an integrated alcohol treatment, and another two described that their alcohol consumption in relation to their psychiatric condition was addressed during their psychological treatment. However, none of the participants recalled having received ‘Guided Self Change’ in step three and participants conveyed the feeling that their AUD was up to themselves to solve on their own.

*Because it is simply not their profession. What they work with. Therefore*,* I haven’t got it* (note: psychological treatment for alcohol-related problems). (Kristin, 50–59 years)

Moreover, one participant described that she had waited a long time to start her psychiatric treatment and was expected to abstain while waiting for, and during the treatment, but received no help to accomplish this.

#### Conversations have the potential to evoke behavior change

Despite the lack of specific alcohol interventions, some participants recalled several conversations about their alcohol use after the initial screening, varying from feedback on blood samples to discussing how their alcohol use affected them. Increased awareness was described to facilitate development of moderation strategies to reduce alcohol use; choosing beverages with lower alcohol content, involving friends and family in non-alcohol related activities such as exercise or asking the social network to support the participant in refusing alcohol. At the same time, social contexts where heavy alcohol use was normalized constituted a barrier to address and reduce alcohol use.

*…the way I drink can vary so it can be like*, *if I hang out with one group then they think that I should drink more but the other group are more understanding that I need to reduce my alcohol use.* (Max, 18–29 years)

### Reflexive monitoring

#### Responses to conditioned psychiatric treatment

As described previously, the participants took actions to become eligible for treatment. The participants described that they felt uncomfortable about these actions and perceived that the alcohol screening lost its purpose. One participant described that her treatment had been conditioned several years ago which still today contributed to her underreporting her alcohol use. Another participant described how he temporarily reduced his alcohol use to ‘pass’ the blood samples.

*.. but the result*,* was that I abstained more during those weeks that I needed to*,* but now when the biomarkers are not taken any longer*,* I can do as I please.* (Fredrik, 30–39 years)

#### A wish for more integrated treatments

A majority of the participants perceived that their alcohol consumption was associated with their psychiatric condition/s, described as either a consequence of their psychiatric condition or as a potential risk factor for developing more severe psychiatric symptoms. The participants expressed a wish for increased knowledge from the clinical staff about the associations between AUD and psychiatric disorders, and more openness to discuss their alcohol consumption.

*I think there should be a larger understanding about how alcohol… and hazardous use is associated to mental illness and what kind of help one can receive at a psychiatric unit like this one. Because to me the association is very clear*,* depending on how I feel it affects how much I drink…* (Matilda, 18–29 years)

More openness was described as key for decreasing the risk for punitive actions, such as cessation of treatment, and instead facilitate a more honest stand from the participant.

Participants discussed the lack of interventions for AUD and wished that alcohol interventions would be offered at the psychiatric units.

*I would like to receive some type of help without maybe ending up at the addiction unit because I know*,* it feels like there is a stigma around…to be marked as addicted. I don´t know how*,* I would just have wished some type of support in this.* (Wilma, 30–36 years)

#### Relevance for the future

As described earlier, the increased awareness about alcohol use, had already led to behavior changes for some participants, while others had realized that they needed to reduce their consumption to prevent future problems. This awareness was described to have the potential to facilitate long-term reductions in alcohol use.

*100%. Since I am so young and now that I assess my habits and have these conversations*,* I think it can*,* that it will help me in the future as well. Definitively. Yes. Long-term.* (Andrea, 18–29 years)

## Discussion

The aim of this study was to investigate how patients in psychiatric outpatient care perceived receiving interventions according to the 15-method, within the context of a large-scale implementation project. The theoretical framework NPT was used to explore the patient’s perspectives of the implementation process.

All participants had been screened for alcohol use according to step one of the 15-method and perceived it as acceptable and leading to increased awareness about their hazardous alcohol consumption. Four participants recalled receiving the assessment in step two, three participants had received pharmacotherapy according to step three but no participant had received the structured psychological treatment according to the ‘Guided Self-Change’ manual, nor had been referred to specialized addiction care. Some participants recognized having had unstructured conversations about alcohol which were perceived to facilitate moderation strategies. A wish to receive more alcohol-interventions at the psychiatric units was expressed. However, in sharp contrast to this wish, participants reported that their psychiatric treatment was conditioned to a non-hazardous alcohol consumption. This suggests that the factors associated with lack of intervention for AUD lie rather on provider or system level than at the patient level. These findings will be discussed more in detail below.

### Alcohol conversations in psychiatric care perceived as acceptable and relevant

The present study found that alcohol screening with the AUDIT questionnaire and brief interventions were seen as both relevant and acceptable in psychiatric care (*coherence*). Participants preferred personalized conversations adapted to their own alcohol use, rather than generic advice on risk levels. Similar call for a personalized approach, has also been made by patients in primary care [36], and is one important aspect to increase *cognitive participation* among patients. Further, the participants perceived a clear association between their psychological health and their alcohol use (*reflexive monitoring*). This is an important finding as previous studies have pointed to a reluctance among health care professionals to raise the topic of alcohol out of fear of offending the patients [16, 17]. The participants also reported that the screening led to increased awareness about their hazardous consumption with potential positive long-term effects with reductions in alcohol use (*collective action*). Increased awareness is of high relevance as AUD, especially as lower severity, is associated with low problem recognition [37], which can contribute to the low rates of treatment seeking for AUD [38]. Screening and brief interventions in psychiatric care has shown to reduce alcohol use [39–41] and reduce re-admissions in psychiatric inpatient care [42]. However, referrals to specialized addiction care have not been shown to increase treatment initiation [40, 43], which emphasizes the need for integrating models such as the 15-method in psychiatric care.

### Low rate of offered alcohol interventions

At the same time as alcohol use was seen as a relevant topic in psychiatric care, the participants described receiving little support from the psychiatric unit to reduce their alcohol use, nor any referral to addiction care.

In Region Stockholm, the implementation of alcohol interventions in psychiatry has been supported by regional guidelines which describes interventions for screening, assessment and treatment. In 2021, the guidelines for AUD were updated and included the 15-method. Despite these efforts, as well as national Swedish guidelines recommending that all patients should be offered pharmacotherapy to reduce or abstain from alcohol use [14], few participants had received AUD pharmacotherapy, and two of those three who received pharmacotherapy described the physician as reluctant to prescribe. It is known that AUD pharmacotherapy is under-utilized [44, 45] where lack of knowledge about AUD pharmacotherapy, perceived low effectiveness and lack of routines are known barriers [44, 46]. Despite a strong preference for psychological treatment for AUD [47], no participant had received the psychological treatment ‘Guided Self-Change’ in the 15-method. In addition, nine of the participants reported an AUDIT score that should trigger referral to specialist addiction care, but none reported being referred.

In relation to collective action, the participants expressed a clear wish to receive more support to reduce their alcohol use, and there was a discrepancy between this wish and the lack of interventions received, leaving the participants with a sense of being alone in changing their alcohol use. The present training initiative in the 15-method and BIC-intervention, targeted factors known to be important in the implementation process such as knowledge, skills to address AUD and local routines [16, 17]. It is therefore puzzling that clinicians still seem hesitant to offer alcohol interventions and indicates that the 15-method has not been successfully embedded and implemented in regular clinical practice. Possibly, the staff had needed a tailored hands-on training focusing on interventions for AUD and co-morbid psychiatric diagnoses, instead of video lectures.

### Wish for openness to discuss both conditions

Alcohol use was seen as a sensitive and difficult subject to talk about, and at the same time, some participants perceived the staff as uncomfortable when approaching the subject. This may be due to staff not wanting to be over-intrusive or stigmatizing [17, 21, 48, 49]. The participants wished to discuss their alcohol use and to be met with greater understanding of the association between alcohol use and psychiatric symptoms. AUD is one of the most stigmatized psychiatric condition [50], which trickles down to clinical practice. Clinicians tend to hold patients with AUD more responsible for their condition compared to patients without AUD and perceive the patients as unmotivated to change [17, 18], which can lead to deteriorating patient-provider relationship and a sub-optimal delivery of health care [51]. The participants in the present study stated that an important part of building a trustful patient-provider relation was to be met with a non-judgmental attitude from the staff when discussing alcohol, confirming previous findings [48]. The present training initiative addressed alcohol use and psychiatric comorbidity, however, our results indicate that these aspects should be further emphasized in future training.

### Conditioned care

Participants described how treatment for their psychiatric disorders was conditioned to low risk levels of alcohol use. Due to fear of losing the psychiatric treatment, the conditioning led to a dishonest patient-provider relationship where participants concealed their actual alcohol consumption. Withdrawal of ongoing treatment has also been reported from England [19], and risks leading to a deterioration of the patients psychiatric health. Previous studies indicate that clinicians perceive patients as dishonest and that they underreport their alcohol consumption [16, 17]. Our findings suggest that in a context where honest reports about alcohol consumption risks leading to punishment, rather than support, it is not surprising that patients are reluctant to disclose their actual alcohol consumption. Further, the conditioning of care was described to lead to superficial reductions in alcohol use, rather than long-term changes. This conditioning of treatment due to high alcohol consumption has not been reported in other settings such as primary care and cardiology [36, 52, 53] and reasons for these differences are unknown. Paternalism, and the use of coercive care, is one aspect of the psychiatric specialty, which might have an impact. Symptoms of psychiatric disorders can make patients unable to take certain decisions and coercive measures can at times be necessary for clinicians to use, but the lines between formal and informal coercion are sometimes blurry and informal coercion has been reported to be commonly used [54, 55]. However, in the present study participants were treated voluntarily and no participant was subject of coercive measures. The conditioning of treatment can be argued to be a type of informal coercion- in sharp contrast to evidence-based interventions for AUD that build on the patient’s own motivation to change [56]. Our findings suggest that these aspects of coercion need to be addressed in future training.

### Strengths and limitations

A strength with this study is the often hard to reach target group – adults with different severities of AUD in psychiatric treatment. Moreover, a large number of psychiatric units were included, representing a variation of psychiatric specialist care.

A limitation is the reliance on self-reports. Due to recall bias, there is a risk that some participants incorrectly remembered when offered alcohol interventions and therefore might have received the interventions before the staff took part in the training project. We tried to minimize these risks by having a structured screening interview to assess eligibility. Furthermore, recall bias may have affected how and what the participants remembered regarding what type of interventions they had been offered and/or received. We tried to minimize this risk by having a semi-structured interview guide, allowing for further discussion around the type of interventions the participant had received. Moreover, the study did not address possible differences between participants with different psychiatric diagnoses.

Those less and more satisfied with the treatment, possibly also reflecting level of motivation to change alcohol use, may be more prone to talk about their experience compared to those that have neutral attitudes, potentially introducing selection bias, threatening internal validity [57] and transferability [58]. Another selection bias may be that the AUDIT questionnaire only measures the last 12 months, excluding people who received help more than 12 months ago. This is however unlikely as training in the 15-method was offered the first units in spring 2021, and recruitment for the current study began in January 2023.

None of the researchers in this study work clinically within psychiatric care, which could influence the interpretation of the results.

Further, this study only investigated the patient perspective and not the clinicians, limiting the conclusions that can be drawn. The clinicians view of the implementation of the 15-method will be elaborated in future studies that investigate the implementation process of the 15-method.

## Conclusions

Patients in psychiatric care perceived screening for alcohol use as acceptable and relevant, and were positive to receive integrated interventions addressing both their alcohol use and their psychiatric disorders in the psychiatric care context. These findings emphasize that the psychiatric treatment context is an important window of opportunity to address both conditions. However, this study suggests that the implementation of AUD treatment might be limited even after a large-scale implementation initiative, and that the psychiatric care often is perceived as conditioned to low risk alcohol use, leaving the patient without support to reduce their alcohol use. Future training and implementation initiatives need to be further tailored to the specific challenges of implementing AUD interventions in psychiatric care, such as tailored hands-on training focusing on interventions for AUD and co-morbid psychiatric diagnoses, practical skills in non-judgmental treatment and reducing the conditioning of care.

## Supplementary Information

Below is the link to the electronic supplementary material.

Supplementary Material 1

## Data Availability

The data underlying this article cannot be shared publicly due to the privacy of the individuals that participated in the study.

## Abbreviations

ADHD: Attention-deficit / hyperactivity disorder
AUD: Alcohol Use Disorder
AUDIT: Alcohol Use Disorder Identification Test
BIC: Building Implementation Capacity
CBT: Cognitive Behavioral Therapy
CDT: Carbohydrate-deficient transferrin
NPT: Normalization Process Theory
PEth: Phosphatidylethanol
PTSD: Post-traumatic Stress Disorder
