# Supplementary material for: A qualitative study of patient’s experiences of receiving alcohol interventions according to the 15-method in psychiatric care
Source: BMC Psychiatry. 2025 Oct 28;25:1031. doi: 10.1186/s12888-025-07553-1 (PMC12570522; doi:10.1186/s12888-025-07553-1)
Supplement: Supplementary file 1 — Supplementary Material 1 [file 12888_2025_7553_MOESM1_ESM.pdf]

Additional file 1. Interview guide following the four construct of NPT – coherence, cognitive participation, collective action, reflexive monitoring.

Background

1. How old are you?
2. What is your gender?
3. What is your country of birth?
4. What is your highest education?
5. What is your occupation?
6. What is the reason you have contact with the psychiatric outpatient unit?
7. How long have you had the contact with the psychiatric outpatient unit?
8. What does the contact consist of? E.g. psychological treatment, pharmacological, neuropsychiatric assessment, and so on.
9. Do you have a psychiatric diagnose? If yes, which is it?
10. What do you think of your alcohol consumption?
11. We are having this interview since you have experienced some type of conversation about alcohol at the psychiatric outpatient unit, can you please tell me about those conversation?

Coherence

How do the patients understand each step of the 15-method? Do they share a common view of the purpose of the different steps? Do they understand how the intervention affects them personally? Do they participants grasp a potential value and benefit of the 15-method?

1. Which profession did you have the conversations with?
2. How many conversations did you have about your alcohol use?
3. What did the conversations about your alcohol use consist of, e.g. AUDIT, conversations about hazardous alcohol use, treatment alternatives? (*Which steps of the 15-method*?)
4. In your opinion, what was the perceived purpose of the conversations?
5. How did you perceive the conversation, e.g. positive/negative? Can you describe more in what way?
6. Was there anything in the conversations that could have been better, be improved or was there anything that you thought was missing?
7. Have the conversations been of benefit for you?
8. How do you perceive that the conversations have affected you in a broader perspective, e.g. your health, work, relations?

Cognitive Participation

How do the patients participate in the 15-method? What builds engagement and keeps the patients motivated to continue to participate?

1. How did you perceive the practical aspects with your contact with the unit, e.g access, booking appointments, testing with biomarkers, prescription?
2. How do you perceive that you have been treated from the staff at the unit in regard to your alcohol habits? Is there anything that could have been better or can improve?

3. Have you talked with other people about your alcohol habits, like friends and family? If yes, how did you perceive those conversations?
4. How does your family, and friends perceive your alcohol habits and your attempts to change it?
5. Have you received help from your family and/or friends in you attempts to change your alcohol habits? In what way? How did you feel about that?

Collective Action
How do the patients make the changes triggered by the different steps in the 15-method? What strategies to they apply to accommodate the new practice, i.e. reduced alcohol use?

1. How was it to talk about your alcohol habits at the psychiatric units, the first time and then broadly speaking?
2. If you have filled out the questionnaire 'Health and alcohol' – how did you find that?
3. Have you received any type of psychological treatment for your alcohol use? If yes: how did you perceive that?
4. Have you received any pharmacotherapy prescribed? If yes: did you use it? How did you perceive that it was to use it, both to take specifically medication for alcohol use and the effect of the medication? Was the effect of the pharmacotherapy monitored? By whom?
5. How was your treatment planned in terms of both your psychiatric diagnoses and your alcohol habits, e.g. different/same conversations, different/same persons, and so on?
6. How did you experience having conversations about your alcohol consumption at the same unit as you have a contact with for your psychiatric symptoms?
7. Have you developed any moderation strategies targeting your alcohol habits? If yes: which ones? Have you perceived them as helpful?
8. Have the conversations been helpful for you to deal with eventual set-backs in changing your alcohol consumption? How?
9. In what way would the treatment possible be different to fit you better?
10. Is there anything that could have been better, improved or that was missing?

Reflexive Monitoring
How do the patients experience that their participation in the 15-method affects them and other people? Do the patients value participating in the 15-method as worthwhile over a longer period of time?

1. In what way have you actively been taken part in the treatment care planning for your alcohol habits?
2. How do you perceive that the conversation about alcohol habits have affected you and other people in your surroundings, e.g. the psychiatric contact, private relations, parenthood, work and so on?
3. Do you believe that the conversation about alcohol will be beneficial for you in a longer perspective? In what way?
